# xtalPiMS: A PiMS-based web application for the management and monitoring of crystallization trials

**DOI:** 10.1016/j.jsb.2011.05.008

**Published:** 2011-08

**Authors:** Ed Daniel, Bill Lin, Jonathan M. Diprose, Susanne L. Griffiths, Chris Morris, Ian M. Berry, Raymond J. Owens, Richard Blake, Keith S. Wilson, David I. Stuart, Robert M. Esnouf

**Affiliations:** a CSED, STFC Daresbury Laboratory, Warrington WA4 4AD, UK; b Division of Structural Biology, University of Oxford, Henry Wellcome Building for Genomic Medicine, Roosevelt Drive, Oxford OX3 7BN, UK; c The Oxford Protein Production Facility UK, Research Complex at Harwell, Rutherford Appleton Laboratory, R92, Harwell Oxford, Didcot OX11 0FA, UK; d York Structural Biology Laboratory, Department of Chemistry, University of York, Heslington, York YO10 5DD, UK; e Diamond Light Source Ltd., Diamond House, Harwell Science and Innovation Campus, Didcot OX11 0DE, UK; f Wellcome Trust Centre for Human Genetics, University of Oxford, Roosevelt Drive, Oxford OX3 7BN, UK

**Keywords:** Laboratory Information Management Systems (LIMS), Protein crystallization, Robotic imagers, Java web application, Data management and databases

## Abstract

A major advance in protein structure determination has been the advent of nanolitre-scale crystallization and (in a high-throughput environment) the development of robotic systems for storing and imaging crystallization trials. Most of these trials are carried out in 96-well (or higher density) plates and managing them is a significant information management challenge. We describe xtalPiMS, a web-based application for the management and monitoring of crystallization trials. xtalPiMS has a user-interface layer based on the standards of the Protein Information Management System (PiMS) and a database layer which links the crystallization trial images to the meta-data associated with a particular crystallization trial. The user interface has been optimized for the efficient monitoring of high-throughput environments with three different automated imagers and work to support a fourth imager is in progress, but it can even be of use without robotics. The database can either be a PiMS database or a legacy database for which a suitable mapping layer has been developed.

## 1 Introduction

There have been rapid advances in the techniques of protein production, crystal growth and structure determination, accelerated in part by structural genomics and structural proteomics initiatives (for recent reviews and overviews of technology platforms see Terwilliger et al., 2009; Weigelt, 2010; Chruszcz et al., 2010; Elsliger et al., 2010; Xiao et al., 2010). A particular aim has been to develop generic techniques which can be applied in parallel, in miniature and on automated platforms. Nowhere have these advances been greater than for protein crystallization by vapor diffusion, where nanolitre-scale pipetting technologies have allowed for at least an order of magnitude reduction in the volume of sample required per crystallization trial coupled with at least an order or magnitude increase in the rate of creation of these trials (reviewed in Berry et al., 2006). This allows many more crystallization trials to be performed, giving a much better sampling of crystallization space and an increase in the number of successful crystallizations. In laboratories with sufficient throughput, a concomitant need for the robotic management of these crystallization trials has been created: storing the trials under controlled conditions coupled with a regular schedule of imaging sessions (also in a controlled environment).

The information management problem associated with the creation of many thousands of crystallization trials is substantial. For laboratories without imaging systems, researchers have to spend long periods at microscope stations and develop more efficient ways of recording the outcomes of trials. For laboratories with access to imaging systems the scale of the problem is much greater, but the scheduled recording of images electronically (*e.g.* as JPEG images) makes it possible to develop management software. Manufacturers of storage and imaging systems usually provide some management software but this may not be suited to academic environments for many reasons, including that

• the user interface may be designed for optimization of known crystallization conditions rather than the discovery of new ones (*i.e.* good for detailed analysis of a few drops rather than quickly scanning many drops for the few good outcomes);
• the software may not handle multiple users in a “crystallization service” model with remote clients and accounting;
• the software may only run on the local control computer;
• the licensing terms or development decisions may restrict the number of concurrent connections; and
• the software itself can be very expensive.

Electronic recording of laboratory information has many potential benefits, and some of these have been discussed elsewhere in an article on the Protein Information Management System (PiMS; Morris et al., 2011). However, as we point out, for low-throughput work on the benchtop (where the benefits are often only felt in the distant future or by other people) adoption of electronic information management is still limited. It was noted that in certain areas electronic recording of data is almost essential: for work that is (i) miniaturized, (ii) parallelized and/or (iii) automated. Protein crystallization is a case in point, exploiting all three approaches, and thus is an excellent candidate for electronic information management.

The Oxford Protein Production Facility (OPPF), established with the support of the Medical Research Council (MRC) in 2001, has been influential in developments in high-throughput crystallization screening. During 2002, the OPPF commissioned a custom-integration of a storage system (The Automation Partnership, Royston, UK) and an imaging station (Veeco-Optimag, San Diego, USA) with capacity for 10,000 SBS-format 96-well plates (Walter et al., 2003,2005; Brown et al., 2003) and developed the PHP-based in-house *Vault* software for web-based management of these trials (Mayo et al., 2005). In complete contrast to the tardy adoption of wet-laboratory Laboratory Information Management Systems (LIMS), there was an (almost) overnight switch to complete dependence on this software across both the OPPF and the Division of Structural Biology (STRUBI) in Oxford. Support was subsequently added for the Oxford lab’s RI1000 and RI182 imagers (Formulatrix, Waltham, USA), but the user interface was not developed and the database schema remained closely aligned to the original storage system. Other web-based viewing software has been developed for crystallization facilities, *e.g.* the CRIMS application primarily in use at EMBL sites (http://embl.fr/htxlab/). CRIMS is a PHP-based application offering a hierarchical view of User → Plate → Thumbnails/Well Images but compared to the *Vault* software it lacks project grouping, zooming, movie/timecourse modes and support for automated analysis.

This article describes xtalPiMS, a web-based application for the management and monitoring of crystallization trials. xtalPiMS has a user-interface layer based on the standards of PiMS and a database layer which links the crystallization trial images to the meta-data associated with a particular crystallization trial. The user interface has been optimized for the efficient monitoring of high-throughput environments. However, it can also be used without robotic imagers: at the Oxford lab an external digital camera has already been linked into xtalPiMS using a customized TWAIN driver to ensure systematic naming of images. The database can either be an instance of a PiMS database or (as is the case at Oxford) a legacy database for which a suitable mapping layer has been developed. xtalPiMS is in service in Oxford, on the UK’s new national protein production facility at the Rutherford-Appleton Laboratory site (RAL; using Formulatrix imagers) and at the York Structural Biology Laboratory (YSBL) to support its BioTom imager. Having already been adapted to support three imagers, work is now underway to support Rhombix (Thermo Scientific) systems at the RAL site, Helsinki and Oulu (Finland) and a feasibility study indicates that xtalPiMS could be integrated with the Minstrel (Rigaku) system. Our model is that xtalPiMS interfaces with the imager databases only to read the crystallization trial meta-data into PiMSdb and then independently scans the image repositories for new imaging sessions. Thus, developers can integrate new imagers with xtalPiMS in a matter of weeks since the dependence on imager software is kept to a minimum and manufacturers have been willing to share either database schemas or APIs, as appropriate.

## 2 Methods

### 2.1 Data models and databases

The PiMS LIMS is based on a sophisticated and complex data model developed from the Protein Production Data Model (PPDM; Pajon et al., 2005) and embodied in the PiMS database (PiMSdb). However, this complexity is largely hidden from the end user (Morris et al., 2011). The model is generalized enough to cope with all eventualities of protein crystallization and a requirement for xtalPiMS was for it to work with PiMSdb and in a complementary manner to PiMS. Thus, PiMS and xtalPiMS can be considered to be two views onto the same data store. Indeed, since they share a common access control mechanism, share many software components and can share the same database, the user need not be aware that they are distinct. The decision to maintain separation was made primarily for developer, administrator and user convenience.

With xtalPiMS development, however, there were additional requirements. First, the software had to be potentially usable with alternative databases, for example the large amount of data already stored in the Oxford crystallization database (PlateDB; Mayo et al., 2005) or that of an equipment manufacturer. Second, xtalPiMS needs to have a flexible software interface to abstract and support different (and even multiple) imagers*.* Third, the large number of crystallization images means that the images themselves are not stored directly within the database, rather they are recorded to an external image store and xtalPiMS has to detect the creation of these image files and link them to the crystallization meta-data. Finally, xtalPiMS must allow users to browse very large numbers of images quickly and ergonomically.

Database performance is a significant issue for xtalPiMS. The requirement not to assume a standard way of working means that PiMSdb is complex and much effort has been spent to optimize its responsiveness for xtalPiMS. However, within one laboratory most crystallization trials are very standardized. For example, the PlateDB database retained simplicity by exploiting the Oxford standard crystallization protocols: only one protein per 96-well plate and sets of standard screens (Walter et al., 2003).

### 2.2 Implementation issues in the crystallization trial browser

**Fig.1:**
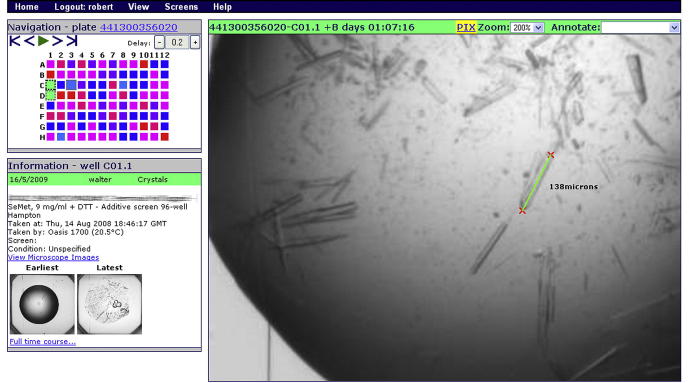
The crystallization trial viewer page in xtalPiMS. The figure shows the plate-based view, the time course-based view has similar functionality but the left-hand side navigation features are appropriate for time courses. The top-left pane shows a schematic view of all wells in the plate colored according to the scores annotated for each drop (the current well is framed in black). The bottom-left pane shows the details of the crystallization trial sample and conditions as well as providing thumbnails of the first and last images in the time course, and links to examine the full time course of images for the current well. The right pane shows a 200%-zoomed crystallization image of the current well and demonstrates the use of the tool to measure crystal size.

The user experience of xtalPiMS is focused on one page, the viewer for a single image of a crystallization trial (an example is shown in Fig. 1). The features of this page from a user-perspective are described in Section 3.2. However, the over-riding design goals have been:

• to allow the user to browse conveniently and rapidly through thousands of images in a single session;
• to offer efficient keyboard shortcuts for commonly used functions;
• to display images as large as possible, and to allow easy zooming and repositioning of images;
• to allow the user easily to locate related images, such as from previous imaging sessions or other plates containing the same protein.

xtalPiMS achieves these goals with heavy dependence on AJAX technologies to allow background fetching of crystallization images from the web server while the user browses those currently loaded. This places some significant load on the client browser and, although generally it works well, some local configuration may be required with certain browsers. The layout of this page automatically adjusts to make maximum use of the browser viewing window, and hence the screen resolution. It is usable in comfort with screen resolutions of 1024 × 768 (XGA) or better.

Although most user time is spent working with one page, xtalPiMS has other pages to allow users to organize viewing sessions and find related images. The set of images making up the time course of a single crystallization trial can be viewed, either as stills or as a movie. Plates set up from a single protein sample can be viewed in turn, and multiple protein samples can be organized into projects with multiple users monitoring the images (see below).

### 2.3 Project-based accounting software

Part of the remit of the OPPF was to provide crystallization as a service – this entails dividing work into projects that are owned and managed by groups of users, keeping track of what plates are set up against each project and accounting usage against groups/projects as appropriate. It also requires xtalPiMS to be able to work efficiently over the web and to control user access to images based on project groups. To achieve this, xtalPiMS at Oxford was modified slightly to enable it to work with an in-house project management package. The model is that projects are created which are owned by a user and for which a certain level of expenditure is authorized. The owning user (usually the principal investigator; PI) can then allow other users permission to register protein samples (and hence create crystallization trials) against this project and to browse the images recorded for these samples. The facility administrator can then “bill” PIs for their actual use, add new “credit” to projects and reassign crystallization trials between projects. However, the ability to control which users are members of each project remains with the PIs themselves. The key stage in making this process effective is that individual protein samples have to be registered against a project before the crystallization setup robots will allow that sample to be used for creating crystallization trial plates. Since the project management package is based on PiMS/xtalPiMS users, a common plate identifier (its barcode) is all that is needed for this package to work with xtalPiMS.

**Fig.2:**
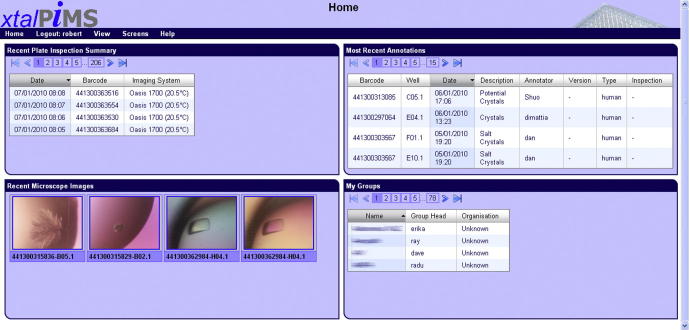
The xtalPiMS home page showing the organization of relevant information into panes. Information in each pane is filtered according to the user’s project membership. The top-left pane tabulates imaging sessions in the past week sorted by date; the top-right pane shows annotations of crystallization images in the past week; the bottom-left pane shows manually recorded images of project crystallization trials in the past week; and the bottom-right pane lists all the user’s projects (this pane is labeled “My Groups” at Oxford for historical reasons).

xtalPiMS works with this software to limit which projects a user is able to see when he/she logs onto xtalPiMS. Fig. 2 shows a typical user home page with the projects they are authorized to see tabulated in a “pane” at the bottom right of the screen. Other relevant data that have been recently updated are shown in the other panes (described in Section 3.1 below). Although not formally part of the xtalPiMS package, this software could also be made available to interested laboratories.

### 2.4 Technical details

xtalPiMS is a high performance Java-based web application developed to work with Java 1.5 or later and makes extensive use of AJAX technologies. As with the rest of the PiMS developments, extensive JUnit testing is done for all software builds. xtalPiMS requires an underlying relational database management system (RDBMS), which can be either Oracle or Postgres if PiMSdb is used. Otherwise, a custom interface layer will need to be developed. The mapping between the object-oriented application and the relational database is handled by Hibernate. xtalPiMS requires no software installation on the client machine – which can be Windows, Linux or Macintosh – and is supported for Internet Explorer 7, Mozilla Firefox 2 and Safari 4 (or later versions).

### 2.5 Installation of xtalPiMS

Upon completion of the PiMS licence agreement, both PiMS and xtalPiMS can be downloaded from http://www.pims-lims.org/. Commercial organizations are required to contact the PiMS team directly for licensing. It is anticipated that most potential xtalPiMS sites will have robotic imagers and dedicated computing for managing the system. The integration between this existing infrastructure and xtalPiMS will require careful planning and may involve some software development. xtalPiMS is a relatively intensive application and one might expect to require a separate web server and database server to ensure good performance. Furthermore, there is likely to be a separate image store which has to be accessible from the client. At the Oxford and RAL sites this is done through an additional web server. xtalPiMS is configured to run in the Apache Tomcat 6 Java application server on either Windows or Linux but could also be configured to run in any Java Servlet v2.5-compliant application server, such as JBoss 5 or Glassfish 2. The database server needs to be running either Postgres 8 or Oracle 10 (or later versions) if the PiMSdb is to be used. The overall installation process, consisting of a series of standard package installs, is relatively straightforward for a competent computer user with some system administration experience.

It is the responsibility of the laboratory to implement suitable disaster recovery procedures and to control access to the system.

## 3 Results and discussion

### 3.1 Using xtalPiMS

Use of xtalPiMS requires no formal training as there are only a small number of pages, which essentially provide a selection mechanism for what is displayed in the main crystallization trial viewer page. Once a researcher has a user account (these are shared between PiMS, xtalPiMS and the project accounting software) and is logged into xtalPiMS he/she is presented with a home page with four panes (Fig. 2). In each pane, the user only gets to see information relating to the projects which they are authorized to view. The top-left pane tabulates recent imaging sessions of project plates; the top-right shows recent annotations of crystallization images in authorized projects by any user; the bottom-left pane shows recent images of project crystallization trials recorded manually on an external microscope; and the bottom-right shows the list of available projects. The user may elect to view a set of images for a plate relating to any imaging session, or a set of images relating to the time course for a single crystallization trial. In either case they end up on the crystallization trial viewer page.

### 3.2 The crystallization trial viewer page

**Fig.3:**
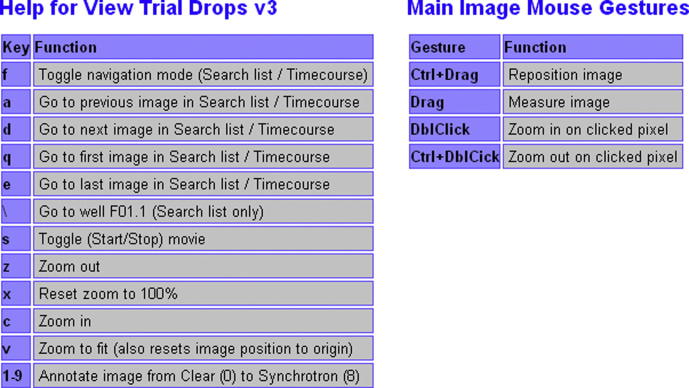
Summary of the mouse gestures and keyboard shortcuts for the crystallization trial viewer page. The heavy use of this page means that an ergonomic design is imperative, and the implementation of appropriate shortcuts is a key feature of this design.

The crystallization trial viewer page (Fig. 1 shows a plate-based view – the time-course view is similar) is kept simple both for performance reasons and to allow the maximum space possible for the main crystallization image. Furthermore, information on the page is updated asynchronously, allowing images to be browsed without the delay of displaying sample information and crystallization conditions (however, when image viewing is paused the correct information is then shown). The mouse can be used for navigation purposes, including the CD-player like controls for the movies of either (a) all wells in a plate or (b) all time steps in a time course. However, there is also an extensive set of keyboard shortcuts and mouse gestures for commonly used functions (Fig. 3).

The most common mode of use is to watch a movie of all wells in a plate, typically at about three images per second. At this speed it is essential that the movie can be interrupted instantaneously when an interesting image is found, for which the keyboard shortcut is very useful. Once an interesting image is found, it can be annotated, using either the drop-down annotation list or by selecting a number on the keyboard. These annotations can be defined by the site administrator, but the set used at Oxford is shown in Table 1. It is these annotations that are shared between users within a project and that are updated on the user home page.

Images can be scaled up for looking at small features. Another useful feature is the measuring tool for checking the sizes of crystals and other objects. By clicking and dragging the mouse over the image a measuring ruler is produced. This ruler automatically rescales as the image is scaled and, within a time course, it is preserved between images. However, it does not persist between user sessions as a permanent annotation.

The client browser, the web server, the database server and the image store all work together to deliver a highly responsive interface to multiple end users simultaneously.

### 3.3 Benefits of electronic management of crystallization trials

Crystallization trials have proven to be ideal for information management systems. The information management requirements for different trials are usually identical; they can be performed in a highly automated manner at high throughput; and they can be effectively monitored remotely using the internet. Above all these considerations, however, using electronic information management makes life significantly easier for the researcher: while a manual microscope may offer slightly better optics and a more interactive viewing experience, sitting in a (cold) crystallization room for hours on end is far from ideal. Sitting at a desk using a web browser allows the researcher to analyse and annotate many more images in a less fatiguing environment. Furthermore, these annotations can be shared over the web, and one can trivially look back at the full history for any particular drop – for example to detect whether crystals are continuing to grow or, conversely, have started to degrade (Esnouf et al., 2006). The sharing of images and annotations is particularly valuable, enabling collaborative working and instant access to crystallization information, for example when working out a suitable cryo-protectant.

### 3.4 Modifications of xtalPiMS for York University and RAL

The Oxford lab uses xtalPiMS exclusively for monitoring crystallization trials, but this version has been adapted to work with PlateDB, the existing legacy crystallization database which now manages >72 million images. In the xtalPiMS installation in York, the generic PiMSdb is used for holding the crystallization meta-data. This installation is based on the ∼500-plate BioStore imaging system (BioTom) and is a lower capacity and throughput operation. Nevertheless, PiMSdb was optimized to enable this installation to provide performance levels comparable with those of the Oxford setup. The York installation provides a very effective platform for managing trials, although there is a lower level of automation at crystallization trial setup time (for example no project-based accounting) requiring more initial manual annotation. Building on the work done for York, the RAL facility also uses xtalPiMS with PiMSdb to schedule and monitor crystallization trials imaged by its three 1000-plate RI1000 imaging systems (Formulatrix, Waltham, USA). Thus, the performance of xtalPiMS on PiMSdb is clearly more than adequate for most laboratories and creates the opportunity for deploying a single information management database covering the whole workflow from target selection to crystallization.

## 4 Conclusions

The potential benefits of electronic recording of laboratory data are clearly demonstrated by its application to crystallization trials. This paper has briefly described the xtalPiMS application, its performance-based design goals; its use for monitoring crystallization experiments and summarized the advantages this software offers researchers. The application is available for installation in any laboratory, but it is especially suited to laboratories with automated storage and imaging systems and a pipeline approach to crystallization. However, any installation will require careful planning and most likely some programming to integrate with the local systems. In the context of the RAL facility, which offers a UK-wide protein production and crystallization service on a site adjacent to the Diamond Light Source, xtalPiMS has been adopted as the crystallization management platform owing to its ability to manage crystallization data and to serve images to many users from across the UK.

## Figures and Tables

**Table 1:** The annotation scheme for crystallization trial images used by Oxford and associated keyboard shortcuts. The York laboratory uses a simpler annotation scheme which does not attempt to distinguish between crystal conditions being the starting point for “optimization”, “crystals” which are good but small/growing and crystals which are suitable for “synchrotron” analysis.

Annotation	Keyboard shortcut
Clear	1
Other	2
Salt crystals	3
Precipitate	4
Needles	5
Potential crystals	6
Optimization	7
Crystals	8
Synchrotron	9
